# Gender Differences in Prevalence and Clinical Correlates of Initial-Treatment and Drug-Naïve Bipolar Disorder Patients With Metabolic Syndrome: A Cross-Sectional Study

**DOI:** 10.31083/AP39112

**Published:** 2025-10-21

**Authors:** Hao Chen, Ye-Hong Chen, Xue-Bing Liu

**Affiliations:** ^1^Affiliated Wuhan Mental Health Center, Tongji Medical College of Huazhong University of Science and Technology, 430012 Wuhan, Hubei, China; ^2^Department of Psychiatry, Wuhan Mental Health Center, 430012 Wuhan, Hubei, China

**Keywords:** bipolar disorder, metabolic syndrome, TT_4_, LDL-C, prevalence

## Abstract

**Background:**

Bipolar disorder (BD) has been studied extensively. However, no studies have investigated gender differences in the prevalence of metabolic syndrome (MetS) in initial-treatment and drug-naïve (ITDN) patients with BD. Therefore, the aim of this study was to investigate gender differences and correlates of MetS in ITDN patients with BD.

**Methods:**

A cross-sectional study of 671 ITDN patients with BD was conducted. Demographic and clinical data were collected. Patients underwent routine serum tests including fasting blood glucose, lipid profile, thyroid function and prolactin tests. Manic, depressive and psychotic symptoms and severity of illness were measured using the Youth Mania Rating Scale (YMRS), the Hamilton Depression Scale (HAMD), the Positive Symptom Scale of the Positive and Negative Symptom Scale (PSS, items P1–P7) and the Clinical Global Impression Scale-Severity of Illness (CGI-SI), respectively.

**Results:**

There was no gender difference in the prevalence of MetS in ITDN patients with BD. Two-way analysis of covariance (ANCOVA) revealed an interaction effect between MetS status and gender for total thyroxine (TT_4_) levels (*p* = 0.005). In addition, multivariable logistic regression analysis revealed that TT_4_ level (odds ratio, OR = 1.426, 95% CI = 1.120–1.817, *p* = 0.004) and PSS score (OR = 1.401, 95% CI = 1.270–1.545, *p* < 0.001) were significantly associated with the MetS in male BD patients; moreover, the low-density lipoprotein cholesterol (LDL-C) level (OR = 2.008, 95% CI = 1.274–3.165, *p* = 0.003) and PSS score (OR = 1.447, 95% CI = 1.316–1.591, *p* < 0.001) were significantly associated with the MetS in female BD patients.

**Conclusion:**

TT_4_ levels and psychotic symptoms were significantly associated with male BD patients with MetS. Furthermore, LDL-C levels and psychotic symptoms were significantly associated with female BD patients with MetS. Particular attention should be given to the early metabolic detection and intervention in male BD patients with high TT_4_ levels and in female BD with high LDL-C levels.

## Main Points

1. The prevalence of metabolic syndrome (MetS) in this study was 16.8% in male bipolar disorder (BD) patients and 19.8% in female BD patients.

2. The interaction between MetS status and gender affects total thyroxine (TT_4_) levels in BD patients.

3. TT_4_ levels and the Positive Symptom Scale of the Positive and Negative Symptom Scale (PSS) score are associated with MetS in male with BD patients.

4. low-density lipoprotein cholesterol (LDL-C) levels and the PSS score are associated with MetS in female with BD patients.

## 1. Introduction

Bipolar disorder (BD) is a chronic, recurrent mental disorder characterized by significant mood fluctuations, including episodes of mania or hypomania and depression [1]. Recent World Mental Health Survey data demonstrate that between 2001 and 2022, the lifetime prevalence of mental illness among 156,331 respondents from 29 countries was 2.5% for males and 2.3% for females [2]. A previous study in China has found that male and female patients with BD have a reduced life expectancy of 6.78 and 7.35 years, respectively [3]. The high incidence, recurrence, disability, and mortality rates of bipolar disorder cause a tremendous social burden [4].

The common definition of metabolic syndrome (MetS) includes symptoms of central obesity, high blood pressure and dyslipidemia [5]. MetS is associated with cardiovascular disease and high mortality in the general population [6]. Moreover, research has indicated that BD patients with MetS exhibit deficiencies in executive function, more pronounced adverse drug reactions, increased hospitalization rates, and diminished overall functionality [7, 8]. A previous study has shown that people with BD have an increased risk of developing MetS compared with the general population [9]. A recent study from China suggested that the prevalence of MetS in BD patients was 33% [10]. One of the important factors that affects BD patients with MetS is the use of antipsychotics, which has been confirmed as a predisposing factor for BD patients with MetS in several studies [11, 12, 13]. Further research revealed that individuals using antipsychotics were 1.72 times more likely to develop MetS than those not using [14]. For BD patients, medications are an important factor contributing to MetS. Notably, factors other than drugs affect the relationship between BD and MetS. The underlying mechanisms involve genetic susceptibility, abnormalities in inflammatory pathways, insulin resistance, and lifestyle factors [15]. BD patients are often more prone to obesity, diabetes mellitus, hypertension, hyperuricemia and ultimately MetS [16, 17, 18, 19]. One study estimated that the prevalence of MetS in drug-naïve BD patients was 33.3% [20]. However, no studies have examined how gender affects the prevalence of MetS in initial-treatment and drug-naïve (ITDN) BD patients.

Gender differences in BD patients with MetS have been extensively studied. For example, females are more sensitive to thyroid function suppression by lithium, which may indirectly affect metabolism [8]. Depressive episodes are more frequent in female patients, and decreased brain-derived neurotrophic factor (BDNF) during depression may exacerbate metabolic abnormalities by inhibiting lipolysis and glucose metabolism [21]. Males are more sensitive to the metabolic side effects of second-generation antipsychotics, possibly due to differences in hormone levels and drug-metabolizing enzyme activity [22]. However, it is not clear whether there are gender differences in the clinical correlates of ITDN BD patients with MetS.

By focusing on ITDN patients, the intrinsic relationship between BD and MetS can be assessed without the confounding influence of psychotropic medications, which are known to significantly affect metabolic parameters. This approach enables a clearer understanding of the metabolic profile associated with BD. Additionally, males and females differ in their hormonal profiles, fat distribution, and metabolic pathways. Understanding these differences may inform gender-specific early screening and intervention strategies for metabolic risk in ITDN BD patients. Therefore, the aim of the present study was to investigate gender differences in the prevalence and correlates of MetS in BD patients.

## 2. Methods

### 2.1 Participants

We conducted a cross-sectional study in Nanyang Fourth People’s Hospital and Wuhan Mental Health Center from January 2016 to December 2024. The study was approved by the Ethics Committee of the Fourth People’s Hospital of Nanyang City (Nanyang Mental Health Centre) with approval code 2016-BHD003 and Wuhan Mental Health Center with approval code KY2022090306. Written informed consent was obtained from all participants and/or their guardians.

Two psychiatrists independently examined all patients using the Mini-International Neuropsychiatric Interview (MINI) standards [23]. Both psychiatrists possess over 10 years of clinical psychiatric experience, formal training in administering the MINI, and prior participation in similar clinical research studies involving diagnostic assessments. A total of 671 ITDN BD patients were recruited from the outpatient and inpatient of Nanyang the Fourth People’s Hospital and Wuhan Mental Health Centre with the following inclusion criteria:

(1) A diagnosis of any form of BD, including bipolar I disorder, bipolar II disorder, manic episodes, depressive episodes, and mixed episodes, was made according to the International Classification of Diseases, 10th edition (ICD-10) [24].

(2) Without prior medication (antipsychotics, mood stabilizers or antidepressants) or physical therapy (transcranial magnetic stimulation, etc.).

(3) Han Chinese individuals between 18 and 60 years of age.

(4) A score of ≥7 on the 17-item Hamilton Depression Scale (HAMD-17) and/or a score of ≥6 on the Youth Mania Rating Scale (YMRS) were given.

The exclusion criteria were as follows:

(1) Any other mental illness such as major depressive disorder, schizophrenia spectrum disorder, personality disorder, substance abuse and dependence, intellectual disability, etc. (assessed using the MINI).

(2) Organic brain diseases, ongoing infections, autoimmune disorders, thyroid disease and other severe physical diseases.

(3) Pregnant or breastfeeding.

### 2.2 Demographic Characteristics

Demographic and clinical variables including age, age of onset, duration of disease (the starting point is when diagnostic criteria are first met), gender, education, marital status, height, and weight were collected for all patients.

### 2.3 Clinical Measurements

Patients were assessed for depressive and manic symptoms and overall illness severity using the HAMD-17 [25], YMRS [26] and the Clinical Global Impression Scale-Severity of Illness (CGI-SI) [27]. In addition, the Positive Symptom Scale of the Positive and Negative Symptom Scale (PSS) (items P1–P7) was used to assess patients’ comorbid psychotic symptoms [28]. This approach aligns with prior methodologies applied in investigations of psychotic symptoms across mood disorders [29]. These assessments were carried out by two attending psychiatrists with the same training, and the results revealed that the correlation coefficients between the scales were consistently greater than 0.8.

### 2.4 Blood Samples and Measurements

Blood samples were collected from all participants between 6:00 AM and 8:00 AM after an overnight fast. Blood samples were collected and transported immediately to the hospital laboratory for testing and measurement at 11:00 AM on the same day. Thyroid-stimulating hormone (TSH), free triiodothyronine (FT_3_), free thyroxine (FT_4_), total triiodothyronine (TT_3_), and total thyroxine (TT_4_) were detected by chemiluminescent immunoassay using a Cobas E610 system (Roche, Basel, Switzerland). Blood creatinine (CRE) and blood urea nitrogen (BUN) were measured using enzymatic methods (CRE: sarcosine oxidase assay; BUN: urease‒glutamate dehydrogenase assay), and blood uric acid (UA) was quantified via the uricase‒peroxidase method, which was performed on a Cobas c501 fully automated biochemistry analyzer (Roche Diagnostics, Basel, Switzerland). Fasting Plasma Glucose (FPG) was detected by the glucose oxidase-peroxidase method, TC was measured using the cholesterol oxidase method, and triglycerides (TG) was determined by the glycerol-3-phosphate oxidase-peroxidase method. High-density lipoprotein cholesterol (HDL-C) and low-density lipoprotein cholesterol (LDL-C) were detected by direct methods, which were performed on a Hitachi 7180 fully-automated biochemical analyzer (Hitachi, Tokyo, Japan). Waist circumference was measured using a flexible tape measure with millimeter accuracy. Participants stood in a natural posture, and measurements were taken at the end of a normal exhalation at the midpoint between the lower margin of the last palpable rib and the top of the iliac crest. Each measurement was repeated twice, and the average value was used for analysis. All samples and clinical data were unknown to the technicians who performed the experiments.

### 2.5 Diagnostic Criteria for MetS

These criteria were customized for Chinese individuals by the Chinese Diabetes Society [30]. MetS is diagnosed when three or more of the following criteria are met: (1) central or abdominal obesity: waist circumference ≥90 cm for men and ≥85 cm for women; (2) hyperglycemia: fasting blood glucose ≥6.10 mmol/L or 2-hour postprandial glucose ≥7.80 mmol/L, or those diagnosed and treated for diabetes mellitus; (3) hypertension: blood pressure ≥130/85 mmHg or those treated for hypertension; (4) elevated fasting TG ≥1.7 mmol/L; and (5) reduced fasting HDL-C <1.04 mmol/L.

### 2.6 Statistical Analysis 

In the present study, we used G*Power 3.1 (Heinrich-Heine-Universität Düsseldorf, Düsseldorf, North Rhine-Westphalia, Germany) to calculate the sample size required for the study [31], with the proportion p1 set at 0.2, proportion p2 set at 0.3, alpha set at 0.05 and the power set at 0.8, calculations demonstrated that sample size of 311 per group. The values of proportion p1, proportion p2 were selected based on previous studies of the prevalence of MetS in male and female BD patients [10, 32, 33]. For descriptive analysis, continuous variables are presented as the means ± SDs, skewed continuous variables are expressed as median (quartiles) and categorical variables are presented as frequencies and percentages. The Mann-Whitney U test was used to compare the skewed continuous variables. The chi-square test and two independent-samples *t*-tests were used to test for differences between groups for categorical and continuous variables, respectively. We performed statistical descriptions and chi-square tests for all BD patients meeting the diagnostic criteria for MetS in each component. Two-way analysis of covariance (ANCOVA) was performed to examine the interaction between MetS (2 levels, with and without MetS) and gender (2 levels, male and female). We included age and disease duration as confounders in the two-way ANCOVA. Univariable logistic regression analysis was used to examine the associations between the study variables and MetS in both male and female patients with BD. We used MetS status as the outcome variable, included the variables that were significant in the univariate analysis in the univariable logistic regression analysis (Enter) one by one, and finally screened out the variables with *p* < 0.05. The screened variables were subsequently included in the multivariable logistic regression analysis. In addition, we included age and disease duration as confounders in the regression model for both groups. The data were analyzed using SPSS version 27.0 software (IBM, Chicago, IL, USA), with statistical significance set at *p* < 0.05 (two-tailed).

## 3. Results

### 3.1 Gender Differences in the Demographic and Clinical Characteristics of BD Patients

The demographic characteristics and clinical variables of male and female BD patients are shown in Table 1. Our results suggest that male BD patients had higher levels of body mass index (BMI) (*t* = 4.497, *p* < 0.001), BUN (*t* = 5.378, *p* < 0.001), CRE (*t* = 9.909, *p* < 0.001) and UA (*t* = 11.355, *p* < 0.001) compared with female BD patients. Female patients had higher levels of TC (*t* = 2.06, *p* = 0.04), HDL-C (*t* = 6.107, *p* < 0.001), TSH (*Z *= 2.239, *p* = 0.025), TT_4_ (*t* = 5.449, *p* < 0.001). In addition, more female BD patients had a spouse (χ^2^ = 22.535, *p* < 0.001). No significant differences in other demographic and clinical characteristics were observed between the two groups. In Table 2, 15.60% of male BD patients had a waist circumference of 90 cm and above, and 37.21% of female BD patients had a waist circumference of 85 cm and above, with a significant difference of male and female BD patients with a waist circumference component that met the diagnostic criteria for the MetS (χ^2^ = 40.035, *p* < 0.001). Low HDL-C was present in 38.23% of male BD patients and 22.67% of female BD patients, with a significant difference (χ^2^ = 19.216, *p* < 0.001).

**Table 1.** **Gender differences in the demographic and clinical characteristics of BD patients**.

Characteristic		Total	Male	Female	*Z*/*t*/χ^2^	*p*-value
		(*n* = 671)	(*n* = 327)	(*n* = 344)		
Age (years)		27 (22–34)	26 (22–32)	28 (22–36)	1.631	0.103
Disease duration (months)		6 (3–11)	5 (3–10)	6 (3–12)	1.732	0.083
Onset age (years)		20 (17–25)	20 (17–25)	20 (17–25)	0.683	0.494
BMI - kg/m^2^		23.63 ± 4.29	24.38 ± 4.34	22.91 ± 4.13	4.497	0.001*
Marital status					22.535	0.001*
	With spouse	217 (32.34%)	77 (23.55%)	140 (40.70%)		
	Without spouse	454 (67.66%)	250 (76.45%)	204 (59.30%)		
Educational background					2.119	0.146
	High school or below	307 (45.75%)	159 (48.62%)	148 (43.02%)		
	Above high school	364 (54.25%)	168 (51.38%)	196 (56.98%)		
MetS					0.973	0.324
	With MetS	123 (18.33%)	55 (16.82%)	68 (19.77%)		
	Without MetS	548 (81.67%)	272 (83.18%)	276 (80.23%)		
MetS components						
	WC (cm)	81.67 ± 8.74	81.90 ± 8.46	81.44 ± 9.02	0.683	0.495
	SBP (mmHg)	124.53 ± 8.33	124.63 ± 7.93	124.40 ± 8.67	0.370	0.711
	DBP (mmHg)	78.35 ± 6.16	78.42 ± 5.89	78.26 ± 6.38	0.350	0.726
	HDL-C (mmol/L)	1.22 ± 0.30	1.15 ± 0.27	1.29 ± 0.31	6.107	0.001*
	TG (mmol/L)	1.35 ± 0.83	1.37 ± 0.78	1.34 ± 0.88	0.474	0.636
	FBG (mmol/L)	5.28 ± 2.64	5.32 ± 3.14	5.24 ± 2.07	0.147	0.701
Renal function						
	BUN (mmol/L)	3.96 ± 1.28	4.23 ± 1.28	3.71 ± 1.22	5.378	0.001*
	CRE (mmol/L)	64.87 ± 24.07	73.73 ± 12.94	56.49 ± 28.72	9.909	0.001*
	UA (mmol/L)	376.88 ± 110.67	422.94 ± 113.38	333.37 ± 88.31	11.355	0.001*
Blood lipids						
	TC (mmol/L)	4.33 ± 1.70	4.20 ± 0.84	4.46 ± 2.22	2.060	0.040
	LDL-C (mmol/L)	2.27 ± 0.74	2.26 ± 0.72	2.28 ± 0.75	0.662	0.508
Thyroid function						
	TSH (uIU/mL)	1.95 (1.25–3.15)	1.85 (1.21–2.81)	2.14 (1.32–3.43)	2.239	0.025
	TT_4_ (ng/mL)	8.02 ± 2.31	7.53 ± 1.80	8.48 ± 2.61	5.449	0.001*
	TT_3_ (ng/mL)	0.98 ± 0.24	0.99 ± 0.22	0.97 ± 0.27	1.263	0.207
	FT_3_ (pmol/L)	3.04 ± 1.33	3.07 ± 0.70	3.01 ± 1.72	0.600	0.549
	FT_4_ (pmol/L)	1.06 ± 0.37	1.06 ± 0.38	1.06 ± 0.37	0.153	0.878
HAMD		18.31 ± 2.70	18.36 ± 2.73	18.25 ± 2.68	0.531	0.596
YMRS		18.03 ± 1.55	18.05 ± 1.56	18.02 ± 1.53	0.189	0.850
CGI-SI		5.49 ± 0.60	5.53 ± 0.60	5.47 ± 0.61	1.308	0.191
PSS		9.33 ± 3.96	9.31 ± 3.97	9.35 ± 3.94	0.131	0.896

Note: MetS, metabolic syndrome; WC, waist circumference; FBG, fasting blood glucose; TG, triglycerides; HDL-C, high density lipoprotein cholesterol; SBP, systolic blood pressure; DBP, diastolic blood pressure; BMI, body mass index; BUN, blood urea nitrogen; CRE, blood creatinine; UA, blood uric acid; TC, total cholesterol; LDL-C, low density lipoprotein cholesterol; TSH, thyroid stimulating hormone; TT_3_, total triiodothyronine; TT_4_, total thyroxine; FT_3_, free triiodothyronine; FT_4_, free tetraiodothyronine; PRL, prolactin; HAMD, Hamilton Depression Scale; YMRS, Young Mania Rating Scale; CGI-SI, Clinical Global Impression Scale - Severity of Illness; PSS, Positive and Negative Syndrome Scale - Positive Symptom Subscale. *p < 0.001.

**Table 2.** **Differences in MetS Components of BD patients**.

MetS Components		Male (*n* = 327)	Female (*n* = 344)	χ ^2^	*p*-value
WC - cm				40.035	0.001*
	male ≥90, female ≥85	51 (15.60%)	128 (37.21%)		
	male <90, female <85	276 (84.40%)	216 (62.79%)		
BP - mmHg				0.069	0.793
	BP ≥130/85	93 (28.44%)	101 (29.36%)		
	BP <130/85	234 (71.56%)	243 (70.64%)		
HDL-C - mmol/L				19.216	0.001*
	HDL-C <1.04	125 (38.23%)	78 (22.67%)		
	HDL-C ≥1.04	202 (61.77%)	266 (77.33%)		
TG - mmol/L				0.833	0.361
	TG ≥1.7	85 (25.99%)	79 (22.97%)		
	TG <1.7	242 (74.0%)	265 (77.03%)		
FBG - mmol/L				0.764	0.382
	FBG ≥6.10	48 (14.68%)	59 (17.15%)		
	FBG <6.10	279 (85.32%)	285 (82.85%)		

Note: FBG, fasting blood glucose; BP, blood pressure. *p < 0.001.

### 3.2 Gender Differences in the Prevalence, Demographic and Clinical Characteristics in BD Patients With MetS

The prevalence of BD with MetS in our study was 16.82% (n = 55/327) in male and 19.77% (n = 68/344) in female. The prevalence of BD with MetS in male patients was similar to that of female patients (16.82% vs 19.77%, χ^2^ = 0.973, *p* = 0.324). As shown in Table 3, a two-way ANCOVA was performed to examine the interaction between MetS and gender, with age and disease duration as covariates. Two-way ANCOVAs which incorporated between-group factors of MetS (2 levels, with MetS and without MetS) and gender (2 levels, male and female) showed that MetS had a significant effect on BMI (*p* < 0.001), waist circumference (WC) (*p* < 0.001), fasting blood glucose (FBG) (*p* = 0.019), systolic blood pressure (SBP) (*p* < 0.001), diastolic blood pressure (DBP) (*p* < 0.001), CRE levels (*p* = 0.015), TG levels (*p* < 0.001), HDL-C levels (*p* < 0.001), LDL-C levels (*p* < 0.001), TT_4_ levels (*p* = 0.017), FT_4_ levels (*p* = 0.010), HAMD scores (*p* = 0.004), CGI-SI scores (*p* = 0.004), PSS scores (*p* < 0.001). These analyses also indicated that there were significant gender differences in BMI (*p* < 0.001), BUN levels (*p* < 0.001), CRE levels (*p* < 0.001), UA levels (*p* < 0.001), HDL-C levels (*p* < 0.001), TT_4_ levels (*p* = 0.017). In addition, there were significant MetS × gender effects on TT_4_ levels (*p* = 0.005). The effect on TT_4_ was analyzed (Analysis of Variance) in male and female BD with or without MetS, respectively. In male patients, TT_4_ levels were higher in BD patients with MetS than in BD patients without MetS (*p* = 0.001). In female patients, there was no significant difference in TT_4_ levels between BD patients with MetS and BD patients without MetS (*p* = 0.826).

**Table 3.** **Demographic and clinical characteristics between BD patients with and without MetS, grouped by gender**.

Characteristic	With MetS (n = 123)		Without MetS (n = 548)		Gender		Subgroup		Gender × subgroup	
	Female (n = 68)	Male (n = 55)	Female (n = 276)	Male (n = 272)	*F* (*p*-value)		*F* (*p*-value)		*F* (*p*-value)	
Onset age - years	22.75 ± 8.75	23.05 ± 8.95	21.54 ± 7.52	22.30 ± 8.20	0.337	0.562	6.917	0.009	3.284	0.070
BMI (kg/m^2^)	24.42 ± 4.94	26.95 ± 4.75	22.53 ± 3.81	23.86 ± 4.07	24.952	0.001*	32.605	0.001*	2.212	0.137
WC (cm)	86.55 ± 8.21	85.30 ± 10.90	80.18 ± 8.77	81.21 ± 7.72	0.000	0.987	37.766	0.001*	1.772	0.184
SBP (mmHg)	130.29 ± 8.78	131.75 ± 9.10	122.94 ± 8.01	123.19 ± 6.85	0.714	0.399	110.344	0.001*	0.591	0.442
DBP (mmHg)	82.93 ± 6.89	83.93 ± 6.73	77.11 ± 5.70	77.31 ± 5.03	0.558	0.455	127.722	0.001*	0.486	0.486
TG (mmol/L)	1.75 ± 0.86	1.73 ± 0.90	1.24 ± 0.85	1.30 ± 0.73	0.249	0.618	31.290	0.001*	0.256	0.613
FBG (mmol/L)	5.78 ± 4.03	5.95 ± 2.56	5.11 ± 1.14	5.19 ± 3.24	0.465	0.495	5.564	0.019	0.031	0.860
BUN (mmol/L)	3.85 ± 1.25	4.07 ± 1.17	3.68 ± 1.21	4.26 ± 1.30	10.481	0.001*	0.158	0.691	2.121	0.146
CRE (mmol/L)	53.20 ± 9.98	67.58 ± 13.56	57.30 ± 31.64	74.93 ± 12.49	50.081	0.001*	5.978	0.015	0.492	0.483
UA (mmol/L)	344.12 ± 73.86	429.26 ± 118.08	330.72 ± 91.45	422.71 ± 112.63	72.339	0.001*	2.074	0.150	0.063	0.802
TC (mmol/L)	4.63 ± 1.08	4.36 ± 1.00	4.42 ± 2.42	4.16 ± 0.80	2.131	0.145	1.061	0.303	0.002	0.960
HDL-C (mmol/L)	1.14 ± 0.27	1.01 ± 0.23	1.32 ± 0.30	1.18 ± 0.27	23.609	0.001*	36.332	0.001*	0.046	0.830
LDL-C (mmol/L)	2.63 ± 0.87	2.46 ± 0.73	2.20 ± 0.69	2.20 ± 0.72	0.806	0.370	19.228	0.001*	1.513	0.219
TSH (uIU/mL)	2.44 ± 2.08	1.78 ± 1.10	2.92 ± 3.70	2.59 ± 4.45	1.114	0.292	3.318	0.069	0.178	0.673
TT_4_ (ng/mL)	8.42 ± 2.17	8.53 ± 2.30	8.50 ± 2.72	7.34 ± 1.62	5.714	0.017	5.675	0.017	7.958	0.005
TT_3_ (ng/mL)	0.96 ± 0.22	1.03 ± 0.27	0.97 ± 0.28	0.98 ± 0.20	3.269	0.071	0.870	0.351	1.713	0.191
FT_3_ (pmol/L)	2.80 ± 0.56	3.22 ± 0.63	3.07 ± 1.90	3.05 ± 0.72	1.931	0.165	0.030	0.863	2.778	0.096
FT_4_ (pmol/L)	1.11 ± 0.32	1.16 ± 0.57	1.05 ± 0.38	1.04 ± 0.32	0.240	0.622	6.593	0.010	0.364	0.546
YMRS	17.81 ± 1.61	17.82 ± 1.93	18.08 ± 1.50	18.09 ± 1.48	0.051	0.822	3.464	0.063	0.000	0.988
HAMD	18.71 ± 2.81	19.15 ± 3.75	18.14 ± 2.64	18.21 ± 2.46	0.665	0.415	8.250	0.004	0.476	0.491
CGI-SI	5.65 ± 0.73	5.62 ± 0.70	5.42 ± 0.57	5.51 ± 0.57	0.233	0.629	8.321	0.004	0.914	0.339
PSS	13.90 ± 5.30	14.56 ± 5.50	8.22 ± 2.49	8.24 ± 2.48	0.597	0.440	358.961	0.001*	1.007	0.316

Note: *p < 0.001.

### 3.3 The Related Factors of BD With MetS in Male Patients

We constructed a multivariable logistic regression of male BD patients with or without MetS as the dependent variable. The variables in the male group were as follows: CRE, LDL-C, TSH, TT_4_, HAMD, and PSS. Age and disease duration included as confounders in multivariable logistic regression. The significant positive predictors for MetS in male BD patients were TT_4_ (OR = 1.426, 95% CI = 1.120–1.817, *p* = 0.004) and PSS (OR = 1.401, 95% CI = 1.270–1.545, *p* < 0.001) (as show in Table 4). Conversely, CRE (OR = 0.942, 95% CI = 0.911–0.975, *p* < 0.001) were significant negative predictors.

**Table 4.** **Multivariable logistic regression analyses of correlates of MetS in male BD patients**.

Variables	B	Std. error	Wald	*p* value	OR	95% confidence interval	
						Lower	Upper
Constant	–5.236	2.104	6.192	0.013	0.005		
Age	0.021	0.024	0.715	0.398	1.021	0.973	1.071
Disease duration	0.057	0.037	2.327	0.127	1.059	0.984	1.139
CRE	–0.059	0.017	11.738	0.001*	0.942	0.911	0.975
LDL-C	0.495	0.281	3.093	0.079	1.640	0.945	2.848
TSH	–0.192	0.169	1.297	0.255	0.825	0.593	1.148
TT_4_	0.355	0.123	8.284	0.004	1.426	1.120	1.817
HAMD	–0.023	0.080	0.083	0.773	0.977	0.836	1.142
PSS	0.337	0.050	45.491	0.001*	1.401	1.270	1.545

Note: CRE, blood creatinine. *p < 0.001.

### 3.4 The Related Factors of BD With MetS in Female Patients

We constructed a multivariable logistic regression of female BD patients with or without MetS as the dependent variable. The variables in the female group were as follows: LDL-C, CGI-SI, and PSS. Age and disease duration included as confounders in multivariable regression models. In Table 5, the significant positive predictors for MetS in female BD patients were LDL-C (OR = 2.008, 95% CI = 1.274–3.165, *p* = 0.003) and PSS (OR = 1.447, 95% CI = 1.316–1.591, *p* < 0.001).

**Table 5.** **Multivariable logistic regression analyses of correlates of MetS in female BD patients**.

Variables	B	Std. error	Wald	*p* value	OR	95% confidence interval	
						Lower	Upper
Constant	–5.114	1.716	8.879	0.003	0.006		
Age	0.024	0.022	1.186	0.276	1.024	0.981	1.069
Disease duration	0.020	0.027	0.534	0.465	1.020	0.967	1.076
LDL-C	0.697	0.232	9.008	0.003	2.008	1.274	3.165
CGI-SI	–0.487	0.313	2.412	0.120	0.615	0.333	1.136
PSS	0.370	0.048	58.144	0.001*	1.447	1.316	1.591

Note: *p < 0.001.

## 4. Discussion

The results of this study suggest that 1. there is no gender difference in the prevalence of MetS in BD patients, 2. TT_4_ levels and the PSS score are associated with MetS in male BD patients 3. LDL-C levels and the PSS score are associated with MetS in female BD patients.

The prevalence of MetS in our study was 16.8% in male BD patients and 19.8% in female BD patients, which is consistent with two studies that reported that the prevalence of MetS in drug-naïve BD patients was 6.5% and 33.3%, respectively [20, 34]. The prevalence of comorbid MetS in BD patients reported in several studies ranged from 27.5% to 53.7% [9, 10, 35]. The focus on drug-naïve patients may explain the lower prevalence of BD with MetS in the present study than in previous studies. Lithium, valproic acid and atypical antipsychotics are associated with weight gain, insulin resistance and dyslipidemia [8, 36]. These findings that drugs are important factors associated with MetS and that studies of unmedicated populations can reveal the metabolic profile of the disease more clearly. There was no significant difference in the prevalence of MetS between male and female BD patients in our study, which is consistent with the results of a previous study conducted in China [33]. Notably, a study of hospitalized patients in Brazil revealed that the prevalence of MetS in female BD patients was 43.6%, which was significantly higher than that in males (20.8%) [32]. Metabolic abnormalities are known to be exacerbated by numerous factors, including chronic inflammatory states, and poor dietary and exercise habits [7]. Indeed, females are more sensitive to thyroid function suppression by lithium, which may indirectly affect metabolism [8]. Patients with BD have abnormal functioning of the hypothalamic‒pituitary‒adrenal (HPA) axis, with elevated cortisol levels promoting abdominal obesity, whereas female patients may have exacerbated HPA axis dysregulation due to estrogen fluctuations (e.g., during menstruation, perimenopause), which may further affect lipid metabolism [37, 38]. Sedentary behavior is prevalent among BD patients, with female patients being less inclined to engage in physical exercise due to mood fluctuations or the sedative effects of medications [39]. While some studies have indicated that patients have a low total caloric intake, the diet is often structured to be high in sugar and saturated fat, exacerbating insulin resistance [40]. While the current study did not observe a gender difference in the prevalence of BD with MetS, the need for future interventions at multiple levels, including drug selection, metabolic monitoring, hormonal regulation, and lifestyle modification, remains paramount.

Our results revealed that more female BD patients than male BD patients have spouses. One explanation is that males with BD often experience manic symptoms, which can be more disruptive to relationships and place a greater caregiving burden on spouses [41]. Symptom severity in patients with BD was significantly associated with hospitalizations, direct medical costs, indirect costs, and quality of life. The more severe the symptoms, the poorer the quality of life, which may indirectly affect marital status [42]. In summary, the marital status of patients diagnosed with BD has been shown to be closely related to the severity of symptoms. When symptoms are mild, marital and family functioning may be more optimal, while in contrast, severe symptoms may lead to exacerbated family conflict and adjustment problems, thereby affecting marital quality. Psychosocial interventions for male patients should thus focus on emotional stabilization and relationship maintenance.

The results of the subsequent 2-way ANCOVA, revealed differences in TT_4_ levels across the different subgroups, with higher TT_4_ levels in male BD patients with MetS than in those without MetS, whereas no such differences were detected in female patients with BD. In the subsequent multivariable logistic regression analysis, we further confirmed that TT_4_ is a correlate for MetS in male BD patients. Recent evidence suggests that impaired thyroid function is strongly associated with the MetS [43, 44]. These studies have reported the relationship of thyroid hormones to MetS and its related components [16, 45, 46, 47]. In previous studies conducted in drug-naïve BD patients, TT_4_ levels were not significantly different from those in the healthy controls [48, 49]. However, several studies have reported a decrease in TT_4_ levels in BD patients treated with mood stabilizers or antidepressants [50, 51, 52]. TT_4_ levels are important regulators of systemic energy metabolism and a level that is either too high or too low can affect the basal metabolic rate, leading to insulin resistance, abnormal fat distribution and metabolic disorders [53, 54, 55], and ultimately the development of MetS. Considering of the extant data, the mechanism of action of TT_4_ levels in MetS may be associated with thyroid dysfunction. Specifically, hypothyroidism has been demonstrated to result in elevated lipids and weight gain, leading to an increased risk of developing MetS [56]. Furthermore, hypothyroidism (e.g., reduced TT_4_ levels) has been demonstrated to be strongly associated with atherosclerosis and insulin resistance [57]. The latter is one of the key pathophysiological mechanisms of the MetS. Therefore, further studies are needed on the mechanism of action of TT_4_ in BD patients with MetS and the gender differences that exist. In the future, thyroid function (especially TT_4_) should be monitored regularly in male BD patients.

The secondary important finding in this study was that LDL-C levels were associated with MetS in females with BD. Few previous studies have been conducted specifically on LDL-C levels in female BD patients with MetS, but the results of the present study are consistent with a variety of previous relevant studies. Jamatia *et al*. [58] reported significantly elevated levels of oxidized LDL-C in patients with MetS. de Melo *et al*. [59] previously reported the existence of a common low-grade inflammatory pathway between BD and MetS, involving the formation of oxidized LDL-C. Oxidized LDL-C is further produced by the peroxidation of LDL-C concentrated in the subendothelium [60]. Oxidized LDL has been demonstrated to promote inflammatory responses and oxidative stress, which in turn can exacerbate the progression of MetS [61]. In patients with MetS, LDL-C is more susceptible to oxidation, leading to vascular endothelial dysfunction and an inflammatory response [62]. In BD patients, a chronic inflammatory state may exacerbate this process and increase cardiovascular disease risk [63]. Decreased levels of estrogen in postmenopausal women lead to increased levels of LDL-C, which can disrupt metabolic levels in the body and ultimately exacerbate MetS [64].

In contrast, no such correlation was observed in male BD patients, a finding that may be attributed to the differing estrogen levels observed between the genders. Liver fat accumulation due to low estrogen levels causes postmenopausal females to have increased LDL-C levels [65]. The reduced function of some estrogen receptors leads to increased serum LDL-C levels when estrogen production is blocked [66, 67, 68]. Moreover, female BD patients are more susceptible to abnormal thyroid function, especially hypothyroidism, and hypothyroidism has been associated with increased LDL-C oxidation capacity, which may indirectly contribute to the pathogenesis of MetS by affecting oxidative stress and inflammatory pathways [69, 70]. van Tienhoven-Wind *et al*. [71] reported a connection between hypothyroidism and elevated LDL-C levels. Regular LDL-C testing is recommended for all female BD patients, especially during the use of antipsychotics. Omega-3 fatty acid supplementation has the potential to reduce LDL-C oxidation and inflammatory markers [63] and may be an adjunctive therapeutic option for BD patients with MetS. More gender-stratified studies are needed to clarify female-specific LDL-C metabolic pathways.

Another major finding of this study is that more severe positive psychiatric symptoms are a correlate for comorbid MetS in both male and female BD patients. To our knowledge, we observed for the first time, a relationship between positive psychiatric symptoms and MetS in both male and female drug-naïve patients with BD. Positive psychotic symptoms often require the use of antipsychotic drugs, which are important mediators of metabolic abnormalities. In particular, atypical antipsychotics are significantly associated with weight gain, insulin resistance, and dyslipidemia [8, 72]. In patients with schizophrenia and major depression, severe psychotic symptoms have been observed as a correlate for MetS [73, 74]. One possible explanation is that psychotic symptoms may lead to increased cortisol through chronic activation of the HPA (i.e., stress) axis, which in turn increases the risk of abdominal fat accumulation, insulin resistance and dyslipidemia [75, 76, 77]. The other explanation is that inflammatory factors, such as IL-6 and TNF-α, have been identified as potential promoters of both psychotic symptoms and metabolic abnormalities, and chronic low-grade inflammation (IL-6 and TNF-α) is often present in patients with BD [78, 79]. This inflammatory environment might promote both psychotic symptoms and metabolic abnormalities. The association between MetS status and positive psychotic symptoms in patients with BD is mediated primarily through side effects of medication. Future longitudinal studies are needed to clarify the direct role of positive symptoms and to integrate metabolic management into BD treatment guidelines to improve prognosis.

## 5. Conclusion

In summary, there was no gender difference in the incidence of MetS among our drug-naïve BD patients. More females than males had spouses. In addition, more severe positive psychiatric symptoms were correlated with MetS in both male and female BD patients, whereas TT_4_ was associated with MetS in only males and LDL-C was associated with MetS in only females. To further understand the association between BD and MetS, and to develop gender-specific diagnostic and therapeutic approaches, it may be important to understand these gender differences. Thus, our comprehensive analysis of male and female patients with drug-naïve BD may further elucidate the interplay between gender and clinical symptoms and contribute to the optimization of BD treatment.

## 6. Limitation

The limitations of our study are as follows. First, the cross-sectional design of this study limits our ability to infer causality. Second, TT_4_ levels are significantly influenced by thyroid-binding globulin (TBG), which was not measured in this study. Third, the number of BD patients with MetS in this study was relatively small, which affects the comprehensiveness and generalizability of the findings. In addition, lifestyle, the female menstrual cycle and other relevant factors that may affect metabolism were not considered in this study. Future studies could investigate causality through multicenter, large-sample longitudinal studies, include more comprehensive clinical characteristics, and expand patient recruitment to improve the generalizability of the findings. By addressing these limitations, it is expected that better preventive and therapeutic strategies will be developed for patients.

## Availability of Data and Materials

The datasets used and analyzed during the current study are available from the corresponding author on reasonable request.
